# Generally Recognized as Safe: Uncertainty Surrounding E-Cigarette Flavoring Safety

**DOI:** 10.3390/ijerph14101274

**Published:** 2017-10-23

**Authors:** Clara G. Sears, Joy L. Hart, Kandi L. Walker, Rose Marie Robertson

**Affiliations:** 1 Department of Communication, University of Louisville, Louisville, KY 40292, USA; clara.sears@louisville.edu (C.G.S.); kandi.walker@louisville.edu (K.L.W.); 2 American Heart Association Tobacco Regulation & Addiction Center, Dallas, TX 75231, USA; rosemarie.robertson@heart.org; 3 American Heart Association, Dallas, TX 75231, USA

**Keywords:** electronic cigarette, e-cigarette, e-cig, GRAS, vape

## Abstract

Despite scientific uncertainty regarding the relative safety of inhaling e-cigarette aerosol and flavorings, some consumers regard the U.S. Food and Drug Administration’s “generally recognized as safe” (GRAS) designation as evidence of flavoring safety. In this study, we assessed how college students’ perceptions of e-cigarette flavoring safety are related to understanding of the GRAS designation. During spring 2017, an online questionnaire was administered to college students. Chi-square *p*-values and multivariable logistic regression were employed to compare perceptions among participants considering e-cigarette flavorings as safe and those considering e-cigarette flavorings to be unsafe. The total sample size was 567 participants. Only 22% knew that GRAS designation meant that a product is safe to ingest, not inhale, inject, or use topically. Of participants who considered flavorings to be GRAS, the majority recognized that the designation meant a product is safe to ingest but also considered it safe to inhale. Although scientific uncertainty on the overall safety of flavorings in e-cigarettes remains, health messaging can educate the public about the GRAS designation and its irrelevance to e-cigarette safety.

## 1. Introduction

Despite scientific uncertainty regarding the safety of inhaling aerosol and flavorant substances from electronic cigarettes (e-cigarettes) relative to inhaling smoke from traditional cigarettes, some consumers and vape shop employees suggest that the U.S. Food and Drug Administration’s (FDA) “generally recognized as safe” (GRAS) designation of constituents provides clear evidence of overall e-cigarette safety [1,2,3,4,5].

Although the Flavor and Extract Manufacturers Association (FEMA) has clarified that the GRAS program only assesses product safety for ingestion in food, not inhalation [6], public confusion appears to continue. In fact, the phrasing of the label itself may be misleading. For example, several public comments on the FDA’s tobacco deeming rule summarized the popular false equivalency, “inhalation of such constituents is harmless because they are designated as ‘generally recognized as safe’ by the FDA” [7].

The objective of this analysis was to assess how college students’ perceptions of e-cigarette flavoring safety are related to understanding of the GRAS designation.

## 2. Methods

After approval from the university’s Institutional Review Board, an online questionnaire was administered to undergraduate students, 18 years or older, at a U.S. Midwestern metropolitan university in spring 2017. Participants were recruited using flyers distributed around campus and in general education courses. The questionnaire assessed perceptions of e-cigarette safety and took approximately 15 min to complete. Tobacco users and non-users between the ages of 18 and 24 years were included in the analysis; participants who had not heard of e-cigarettes were excluded. Data were collected in REDCap and analyzed using SAS 9.4 (SAS Institute Inc., Cary, NC, USA).

Perceptions of the safety of e-cigarette flavorings were assessed by participants’ responses to the statement, “It is safe to inhale flavorings in electronic cigarettes”. The response options were dichotomized into: Not Safe and Safe. Chi-square *p*-values were calculated to compare perceptions of flavoring safety to other questions regarding the GRAS designation and perception of e-cigarette safety (Table 1). E-cigarette use was categorized as Never used, Tried, and Current use. Current use was defined as any use of an e-cigarette in the past 30 days. Tried was defined as previous use of e-cigarettes, but no use in the past 30 days. Never used was defined as never having used, or even tried, an e-cigarette. Participants that did not want to disclose e-cigarette use were categorized as Never used (*n* = 14). Using stepwise procedures, covariates related to the primary outcome, perception of flavoring safety (significance level of *p* < 0.20), were considered for inclusion in a Multivariable Logistic Regression model. Among participants who considered flavorings to be GRAS, understanding of the GRAS designation was further assessed (Table 2).

## 3. Results

Overall, the total sample size for this analysis was 567 participants. Individuals were excluded because they had not heard of e-cigarettes (*n* = 40), were outside the age range (*n* = 22), or had missing data (*n* = 23). Approximately 67% of participants were females and the median age of participants was 20 years (range = 18–24), with a mean of 20.2 years (standard deviation = 1.5). More current e-cigarette users perceived flavorings in e-cigarettes as safe to inhale (*p* = 0.03).

Approximately 53% did not disagree with the statement, “It is safe to inhale flavorings in e-cigarettes”. Overall, only 22% of participants knew that GRAS designation meant that a product is safe to ingest, not inhale, inject, or use topically. Among participants who considered flavorings to be GRAS, the majority recognized that a GRAS substance is safe to ingest but also considered it safe to inhale (Table 2).

In a Multivariable Logistic Regression model adjusting for overall perception of e-cigarette safety, participants considering flavorings as GRAS were more likely to believe that it is safe to inhale flavorings in e-cigarettes when compared to participants disagreeing with the statement “it is safe to inhale flavorings” (OR = 6.1; 95% CI = (3.9, 9.6)).

## 4. Discussion

E-cigarette use among young adults is a public health concern, and targeted marketing practices promoting misconceptions about the safety of e-cigarettes may lead to a broader acceptance and use of the novel tobacco product [8].

Although scientific uncertainty on the safety of e-cigarette aerosol constituents relative to traditional tobacco smoke remains, health messaging can educate the public about the GRAS designation and its irrelevance to e-cigarette safety. In particular, the FDA and similar bodies should consider developing educational campaigns to combat the misapplication of the GRAS designation to e-cigarette flavorings and constituents.

## 5. Conclusions

The findings of this study indicate that only approximately one-fifth of college students surveyed understand that GRAS designation is limited to ingestion and that the remaining nearly four-fifths misinterpret GRAS designation. Public misunderstanding of GRAS designation perpetuates views that e-cigarette flavorings are safe, when much scientific testing remains prior to fully understanding the health effects of these products.

## Figures and Tables

**Table 1:** Perception of e-cigarette flavoring safety- % (*n*).

Variable	Not Safe 46.6 (264)	Safe 53.4 (303)	Total 100 (567)	*p*-Value
**Gender**				0.05
*Female*	71 (186)	64 (192)	67 (378)	
*Male*	29 (75)	36 (110)	33 (185)	
**Age**				0.68
*20 years or less*	56 (147)	57 (174)	57 (321)	
*21 years or more*	44 (117)	43 (129)	43 (246)	
**E-cigarette Use**				0.03
*Never used*	67 (177)	60 (183)	63 (360)	0.43 ^a^
*Tried*	29 (76)	30 (91)	30 (167)	0.009 ^b^
*Current use*	4 (11)	10 (29)	7 (40)	
**Electronic cigarettes are safe**				<0.0001
*Disagree*	88 (231)	54 (163)	70 (394)	
*Do not disagree*	12 (33)	46 (140)	30 (173)	
**Flavorings are generally recognized as safe by the FDA**				<0.0001
*Disagree*	33 (88)	9 (26)	20 (114)	
*Do not disagree*	67 (176)	91 (277)	80 (453)	

Values presented in table are column percentage (count). Significance determined at *p* < 0.05. Response options were formatted as Likert scale (Strongly Disagree, Disagree, Neutral, Agree, Strongly Agree) and dichotomized into categories for analysis: Not Safe/Disagree (Strongly Disagree and Disagree) and Safe/Do not disagree (response categories: Neutral, Agree, Strongly Agree). ^a^ *p*-value for comparison between Tried and Never used; ^b^ *p*-value for comparison between Current use and Never used.

**Table 2:** Understanding of “generally recognized as safe” (GRAS) among people who consider flavorings to be GRAS- % (*n*).

	If a Substance Is GRAS, It Is Safe to Inhale			
If a Substance Is GRAS, It Is Safe to Ingest	No 26 (116)	Yes 74 (337)	Total 100 (453)	*p*-Value
*No*	28 (33)	42 (140)	26 (116)	0.01 ^a^
*Yes*	72 (83)	58 (197)	74 (337)	

Values presented in table are column percentage (count). ^a^ Chi-square test *p*-value.
